# Automating licking bias correction in a two-choice delayed match-to-sample task to accelerate learning

**DOI:** 10.1038/s41598-023-49862-z

**Published:** 2023-12-20

**Authors:** Jongrok Do, Min Whan Jung, Doyun Lee

**Affiliations:** 1 Department of Biological Sciences, Korea Advanced Institute of Science and Technology, Daejeon, 34141 Republic of Korea; 2 Center for Cognition and Sociality, Institute for Basic Science, Daejeon, 34126 Republic of Korea; 3 Center for Synaptic Brain Dysfunctions, Institute for Basic Science, Daejeon, 34141 Republic of Korea

**Keywords:** Biological techniques, Neuroscience

## Abstract

Animals often display choice bias, or a preference for one option over the others, which can significantly impede learning new tasks. Delayed match-to-sample (DMS) tasks with two-alternative choices of lickports on the left and right have been widely used to study sensory processing, working memory, and associative memory in head-fixed animals. However, extensive training time, primarily due to the animals' biased licking responses, limits their practical utility. Here, we present the implementation of an automated side bias correction system in an olfactory DMS task, where the lickport positions and the ratio of left- and right-rewarded trials are dynamically adjusted to counterbalance mouse’s biased licking responses during training. The correction algorithm moves the preferred lickport farther away from the mouse’s mouth and the non-preferred lickport closer, while also increasing the proportion of non-preferred side trials when biased licking occurs. We found that adjusting lickport distances and the proportions of left- versus right-rewarded trials effectively reduces the mouse’s side bias. Further analyses reveal that these adjustments also correlate with subsequent improvements in behavioral performance. Our findings suggest that the automated side bias correction system is a valuable tool for enhancing the applicability of behavioral tasks involving two-alternative lickport choices.

## Introduction

Operant conditioning, in which animals learn to associate stimuli with behaviors that lead to desired outcomes, has been widely used for understanding animal behavior and cognition. By manipulating stimuli and outcomes, operant conditioning enables researchers to study how animals make perceptual decisions^1^. Animals often exhibit biases in their choices, favoring one alternative over the others, due to a bias in the sensory or decisional processes^2^. This phenomenon, known as choice bias^3^, has been widely observed across species and remains stable even though it leads to a decrease in the total amount of reward recieved^3,4^. Choice biases serve as efficient behavioral strategies for discriminating perceptually similar stimuli^5^, and can be advantageous for learning and increase survival chances^5,6^. However, these choice biases hinder animals when they are learning the association between perceptual decisions and behavioral responses. This is because choice biases, which are highly correlated with error rates^5^, reduce the opportunities for animals to receive reinforcement and impedes the learning process.

Choice biases are also influenced by the asymmetry between available choice alternatives. Go/No-Go tasks, which are widely employed behavioral paradigms, are particularly vulnerable to choice biases owing to differences in motivational level, task engagement (attention), and reward expectations between choice options^7–9^. For example, in a Lick/No-lick task, one version of the Go/No-Go paradigm, mice face two behavioral options: to lick or refrain from licking a lickport^9^. Each option is associated with either a water reward or no reward, resulting in a significant asymmetry in reward availability. Mice display an intrinsic bias toward licking to obtain the reward^10^, making it challenging to differentiate between active licking following correct perceptual decisions and indiscriminate licking driven by compulsive reward seeking, as well as between active refraining from licking and loss of motivation^9^.

To address the disadvantages of the Go/No-Go task, some researchers have adopted a two-alternative choice paradigm in which the choices are relatively equal. These paradigms involve symmetric choices such as licking the left or right port^11–37^, steering a wheel to the left or right^38–45^, moving a treadmill to the left or right or rotating a trackball to the left or right^46–48^. These symmetric designs ensure comparable levels of motivation, task engagement, and reward expectations for both choices. Additionally, missed trials can be interpreted as a loss of task engagement, whereas error trials can be attributed to perceptual or decision errors^8,9^. However, compared with the Go/No-Go paradigms, the two-alternative choice tasks typically require significantly more time to train mice to meet the high-performance criterion. For example, olfactory delayed non-match-to-sample tasks take about a week for mice to learn when one lickport was used^49,50^. However, it requires substantially longer training when combined with two-alternative lickport choices^20^. Prolonged training in two-alternative choice tasks increases the workload for researchers and hampers the efficient acquisition of a sufficient amount of data.

To overcome the challenges associated with lengthy training periods, researchers have implemented algorithmic strategies to expedite the learning process. One widely used approach is to determine the next trial type based on the animal’s current behavior. A typical example is the correction procedure, in which incorrect choices are followed by the repetition of the same trial type until the correct response is made. This reinforces the association between the reward-associated stimulus and the appropriate action^41,51–71^. Another example is continuously adjusting the probability of the left- and right-rewarded trials based on the estimated side bias derived from recent behavioral responses. Nevertheless, despite the implementation of these strategies, the physical accessibility of the choice options remains equal for animals, limiting the efficacy of these strategies.

There are also strategies that manipulate the physical accessibility of different choices. In Go/No-Go tasks, mice often exhibit a bias toward licking^10^. Positioning the lickport away from the mouse’s mouth effectively suppresses compulsive licking in No-Go trials because it requires more effort for the mouse to lick the lickport. In two-alternative choice paradigms with left and right lickports, placing the preferred lickport laterally away from the mouth helps to balance the mice’s preferences and encourages equal engagement with both lickports^10^. However, manually adjusting lickport positions is not feasible on a trial-by-trial basis, which poses challenges in effectively addressing dynamic changes in the side preference. Moreover, if the lickport position is not promptly returned to a symmetrical configuration after resolving side bias, it may induce a bias toward the opposite side. Additionally, the manual adjustment of lickport positions can introduce potential human bias into the behavioral results.

To overcome the limitations of manual correction, implementation of automatic lickport adjustment systems is an ideal solution. A previous study introduced a motor-controlled lickport for olfactory-based tasks in head-fixed mice^72^. This system facilitated the learning process by moving the lickport near the mouse’s mouth at the start of the response window in each trial, effectively encouraging the mice to lick. Although this approach adopted a program-controlled lickport position adjustment on a trial-by-trial basis, its primary focus was on teaching mice to associate the response window with reward delivery, specifically teaching them when to lick. However, it did not directly assist the animals to associate between stimulus combinations and corresponding behavioral responses. To the best of our knowledge, no study has employed a program-controlled lickport position adjustment on a trial-by-trial basis to assist mice to associate stimulus combinations and behavioral responses.

In this study, we developed a novel automatic training system with computer program-controlled movable lickports. This system enables the dynamic adjustment of lickport positions and the ratio of left- and right-rewarded trials based on the animals’ behavioral performance. Specifically, we applied this system to the olfactory-based delayed match-to-sample (DMS) task with two-alternative choices, a task known to require prolonged training for mice compared with paradigms with asymmetric Go/No-Go choice options^20,49,50^. Our findings demonstrate that our algorithmic approach significantly reduces the training time required for mice to learn the DMS task. Through comprehensive analyses, we reveal that the adaptive adjustment of lickport positions and trial ratios effectively mitigates side biases and enhances behavioral performance in the DMS task. These findings emphasize the efficacy of our automatic training system in promoting research on perceptual decision-making and working memory.

## Methods

### Animals

Eleven male C57BL/6J wild-type mice (The Jackson Laboratory; 2–3 months old) were used for the behavioral experiments. Following the head bar surgery, the mice were individually housed in separate cages. They were maintained under a 12-h light/dark cycle (humidity: 40%, temperature: 22 °C) with food and water ad libitum. Before the onset of handling, the mice were subjected to a water restriction schedule, receiving approximately 1 mL of water/day. All procedures used in this study were approved by the Institutional Animal Care and Use Committee of the Institute for Basic Science (Daejeon, South Korea). All experiments were performed in accordance with the institutional guidelines. All authors complied with the ARRIVE guidelines.

### Surgery

The mice were anesthetized using isoflurane (5% for induction, 1%–2% for maintenance) and secured in a stereotaxic frame (SR-6M-HT; Narishige, Japan) while maintaining the body temperature at 37 °C using a heating pad (DC temperature control system, FHC Inc., Bowdoin, ME, USA). An ophthalmic ointment was applied to the eyes to prevent dryness during surgery. Before making an incision on the scalp, 0.5% bupivacaine in saline was administered for local anesthesia. The scalp and periosteum covering the dorsal surface of the skull were then removed, and a T-shaped stainless-steel head bar was firmly attached to the skull using light-cured dental composite and dental acrylic. After at least 7 days of recovery, animals proceeded to the behavioral training procedures.

### Behavioral apparatus

**Figure 1:**
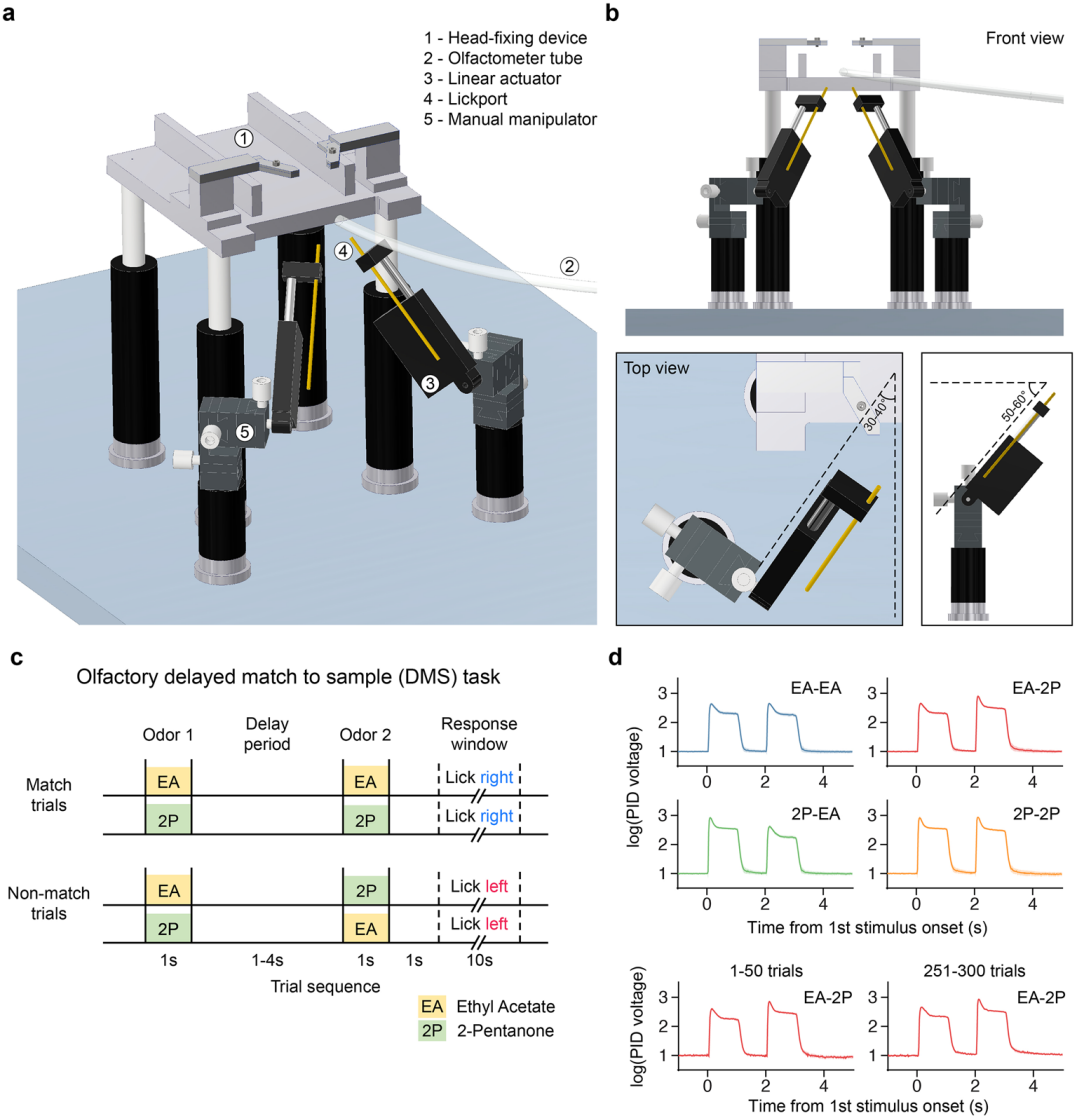
Behavioral apparatus and delayed match to sample (DMS) task paradigm. (**a**) Schematic representation of the behavioral apparatus. (**b**) Top: Front view of the behavioral apparatus. Bottom: Angular relationship between individual devices. (**c**) Schematic of the DMS trial. (**d**) Changes in the odor concentration during DMS trials as measured using a photoionization detector (PID). Top: Time course of the odor delivery for four different stimulus combinations. Bottom: Odor concentration for the first and last 50 trials for an example combination (EA-2P) in a session.

The experimental apparatus was constructed within a soundproof box measuring 64 cm (w) × 64 cm (l) × 60 cm (h). A head-fixing device for the subject mouse was positioned at the center of the box. Two linear actuators (12Lf-12PT-27; IR Robot, South Korea) were mounted on each manual three-axis micromanipulator on the left and right sides of the mouse’s head (Fig. 1a). Then, a lickport was attached at the end of the piston rod of each linear actuator using a custom-made adaptor. The lickports were positioned parallel to the travel axis of the linear actuators and at an angle to the vertical axis when viewed from the front and side (Fig. 1b). This arrangement required the mice to adapt the extension distance of their tongues without altering the angles at which they stuck out their tongues, in response to the changing positions of the lickports. The lickport, made of a stainless-steel tube (diameter: 1.3 mm), also served as a lick detection sensor^73^. Water rewards were delivered via a tube connecting the lickport to a water reservoir (a 10 ml syringe) placed 50 cm above the lickport, utilizing gravity. A solenoid valve (161T011; NResearch Inc., NJ, USA) was used to control the delivery of rewards. The valves and motors were controlled by an Arduino board (Arduino Mega 2560 R3). A graphical user interface (GUI) created with the MegunoLink Pro (Hamilton, New Zealand) was used to control task parameters and display task progress. A monochrome camera (BFLY-U3-03S2M-CS, FLIR, Canada) was positioned in front of the subject mice to monitor their licking behavior.

Odorants were delivered using a custom-made olfactometer adapted from a previous study^72^, which was designed to ensure reliable and thorough mixing of pure air with odorants, and to maintain a stable odor concentration throughout the session, spanning over 300 trials (Fig. 1d). Pure air was continuously delivered to the mice at a constant flow rate of 1.0 L/min during the entire session. One of two odorants (ethyl acetate (EA) and 2-pentanone (2P)) was mixed with air during the odor presentation period^49^. The volume ratio of these odorants in the pure air was 5%. Three-way solenoid valves (LHDA0533115H; Lee Company, USA) were used to control the flow of air and odorants (one hole plugged). The odor mixtures were presented to the mice through a transparent polyurethane tube (inner diameter: 4 mm), which was positioned 1 cm away from their noses. Rapid changes in the concentration of odors delivered to the mice and the stability of the peak odor concentration across trials were verified using a photoionization detector (200B; Aurora Scientific, Canada; Fig. 1d).

### Delayed match-to-sample task

In each trial, the mice were presented with odors twice, with each presentation lasting for 1 s. The delay between the two odor presentations varied depending on the training phase, ranging from 1 to 4 s (Fig. 1c). One second after the offset of the second odor presentation, the mice were allowed to report their choice by licking one of the two lickports within a fixed response window (30 s for the habituation phase and 10 s for the training phase). In the nonmatch condition, where the first and second odors differed, licking the right lickport triggered a water reward (2 μL), whereas when in the match condition, wherein the two odors were the same, licking the left lickport triggered a water reward.

The lickports were only accessible to the subject mice during the response window, discouraging unnecessary licking during the stimulus presentation as well as the delay period. This was achieved by adjusting the positions of the lickports using the linear actuators, which moved them between the active position (within reach of the tongue) and the inactive position (5 mm away from the active position, keeping them out of the reach of the tongue) at a travel speed of 25 mm/s. When the mice licked the lickport on the correct side, the lickport remained in place for 1 s, allowing the mice sufficient time to consume the water reward. However, if the mice licked the incorrect lickport, both lickports retracted to the inactive position, accompanied by a brief feedback sound (200 ms, 4 kHz, 50 dB, without shielding) emitted from a mini speaker positioned 100 mm away from the mice. Each trial was followed by a nine-second inter-trial interval.

### Automated side bias correction algorithm

**Figure 2:**
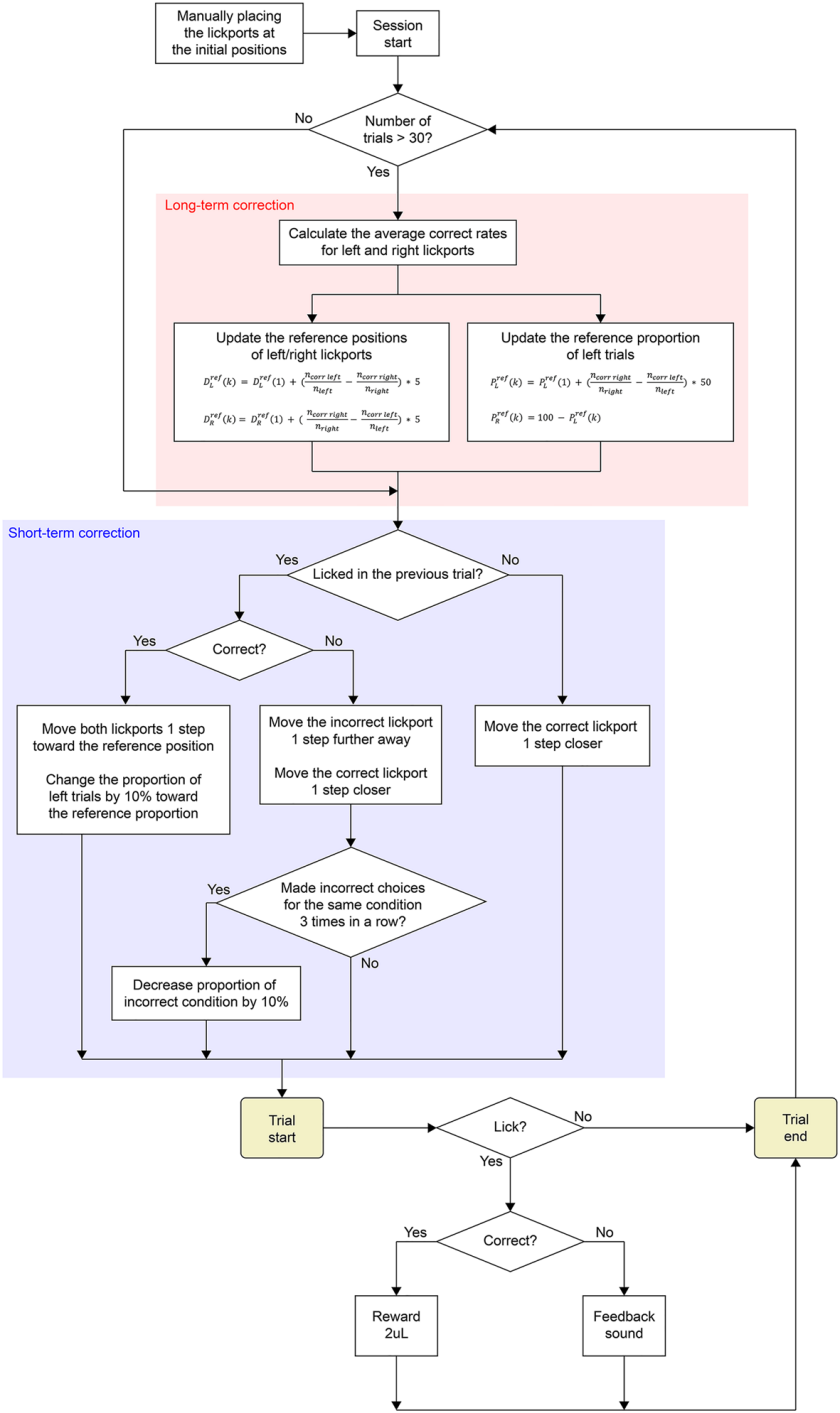
Flow chart of the automated side preference correction algorithm.

The majority of mice used in this study displayed a bias toward one side^9^. Throughout the training process, we continuously monitored the side bias exhibited by the mice. If a bias toward one lickport was detected, we adjusted the positions of both lickports. The lickport on the preferred side was moved farther away from the mice, whereas that on the nonpreferred side was brought closer. Additionally, we modified the proportion of the left-rewarded (match) and right-rewarded (nonmatch) trials to increase the probability of rewarding the nonpreferred side (Fig. 2). These adjustments were implemented based on the evaluation of the mice’s performance during two different time periods: long-term and short-term corrections. The long-term correction (Fig. 2, red box) considered the side bias evaluated across all the trials performed by the mice, whereas the short-term correction (Fig. 2, blue box) considered only the most recent trials as the basis for adjustments.

#### Parameter definition

${D}_{L}(k)$, ${D}_{R}(k)$: Active positions of the left (L) and right (R) lickports in trial *k.*

${D}_{L}^{ref}(k)$, ${D}_{R}^{ref}(k)$: Reference positions of the left (L) and right (R) lickports in trial *k.*

${P}_{L}(k)$, ${P}_{R}(k)$: Probability of trial *k* to be a left (L)- or right (R)-rewarded trial.

${P}_{L}^{ref}(k)$, ${P}_{R}^{ref}(k)$: Reference proportion of left (L)- and right (R)-rewarded trial in trial *k.*

#### Long-term correction

Throughout a session, animals often displayed a consistent preference for one side, which was evidenced in their higher accuracy for the trials of the preferred side and lower accuracy for the trials of the nonpreferred side. The long-term correction aimed to reduce the overall side bias within each session (Fig. 2, red box). Before a session began, the initial active positions of the lickports were set at the distance where the mice could comfortably lick, approximately 5–10 mm from the mouth. These positions were defined as the *initial reference position* (${D}_{L}^{ref}(1)$ and ${D}_{R}^{ref}(1)$). A higher value indicated a greater distance from the mouth. Additionally, we defined ${P}_{L}^{ref}(1)$ as the *initial reference proportion of left-rewarded trials*, which was set to 50% at the beginning of a session. The proportion of left-rewarded trials represented the probability of the current trial being a left-rewarded (non-match) trial. The reference lickport positions (${D}_{L}^{ref}(k)$ and ${D}_{R}^{ref}(k)$) and the reference proportions of left- and right-rewarded trials (${P}_{L}^{ref}(k)$ and ${P}_{R}^{ref}(k)$) were adjusted based on an estimation of the side bias using the following equations:

$$D_{L}^{ref}(k) = D_{L}^{ref}(1) + \left( {\frac{{n_{corr\_left} }}{{n_{left} }} - \frac{{n_{corr\_right} }}{{n_{right} }}} \right) * 5$$

$$D_{R}^{ref}(k) = D_{R}^{ref}(1) + \left( { \frac{{n_{corr \_right} }}{{n_{right} }} - \frac{{n_{corr \_left} }}{{n_{left} }}} \right) * 5$$

$$P_{L}^{ref}(k) = P_{L}^{ref}(1) + \left( {\frac{{n_{corr \_right} }}{{n_{right} }} - \frac{{n_{corr \_left} }}{{n_{left} }}} \right) * 50$$

$$P_{R}^{ref}(k) = 100 - P_{L}^{ref}(k)$$

where ${n}_{left}$ and ${n}_{right}$ are the total number of left- (non-match) and right-rewarded (match) trials, respectively, up until trial *k* − 1; ${n}_{corr \_left}$ and ${n}_{corr \_right}$ are the number of correct answers for the left- and right-rewarded trials, respectively, during trials 1 to *k* − 1. ${D}_{L}^{ref}(k)$ and ${D}_{R}^{ref}(k)$ varied in increment of 1, ranging from − 5 to + 5 relative to ${D}_{L}^{ref}(1)$ and ${D}_{R}^{ref}(1)$, where a value of 1 corresponded to a step size of 0.25 mm, as determined empirically for the linear actuator. In addition, ${P}_{L}^{ref}(k)$ could vary by 10%, ranging from 0 to 100%. The long-term correction parameters (${D}_{L}^{ref}(k)$, ${D}_{R}^{ref}(k)$, and ${P}_{L}^{ref}(k)$) were updated after every trial (Fig. 2, red box), but only after completing the initial 30 trials to prevent extreme fluctuations in the correction parameters due to an insufficient number of trials.

Notably, the long-term correction did not directly adjust the actual lickport positions (${D}_{L}(k)$ and ${D}_{R}(k)$) and the proportion of left- and right-rewarded trials (${P}_{L}(k)$ and ${P}_{R}(k)$). It only set the direction of changes for the short-term correction described below.

#### Short-term correction

While the long-term correction addressed the overall side preference throughout a session, it did not capture the transient fluctuation in side bias and behavioral performance in the range of a few trials. To immediately counteract the transient fluctuation of side bias, we adjusted the lickport positions and the trial ratio according to the behavioral performance during the recent few trials (Fig. 2, blue box). This short-term correction was applied from the beginning of each session.

1. If the mice incorrectly chose a lickport (e.g., left lickport) in trial *k*−1, the position of that lickport was adjusted one step (0.25 mm) farther away (${D}_{L}(k)$ = ${D}_{L}(k-1)+1$), while the position of the other lickport was adjusted one step closer (${D}_{R}(k)$ = ${D}_{R}(k-1)-1$).
2. If the mice chose the correct lickport in trial *k*−1, the positions of both lickports were adjusted one step closer to their reference positions (${D}_{L}^{ref}(k)$ and ${D}_{R}^{ref}(k)$).
3. If the mice made three consecutive incorrect choices for the same side (e.g., choosing the right side in left-rewarded trials three consecutive times without considering choices in the intervening right-rewarded trials), the proportion of trials for that side increased by 10% (e.g., ${P}_{L}(k)$ = ${P}_{L}(k-1)+10$).
4. If the mice made a correct choice in trial *k*−1, the proportions of the left- and right-rewarded trials (${P}_{L}(k)$ and ${P}_{R}(k)$) were shifted by 10% toward the reference proportions (${P}_{L}^{ref}(k)$ and ${P}_{R}^{ref}(k)$).

The short-term correction algorithm adjusted the current lickport positions (${D}_{L}(k)$ and ${D}_{R}(k)$) and current proportions of left- and right-rewarded trials (${P}_{L}(k)$ and ${P}_{R}(k)$) around the reference positions (${D}_{L}^{ref}(k)$ and ${D}_{R}^{ref}(k)$) and the reference proportions (${P}_{L}^{ref}(k)$ and ${P}_{R}^{ref}(k)$), respectively.

### Training protocol

**Figure 3:**
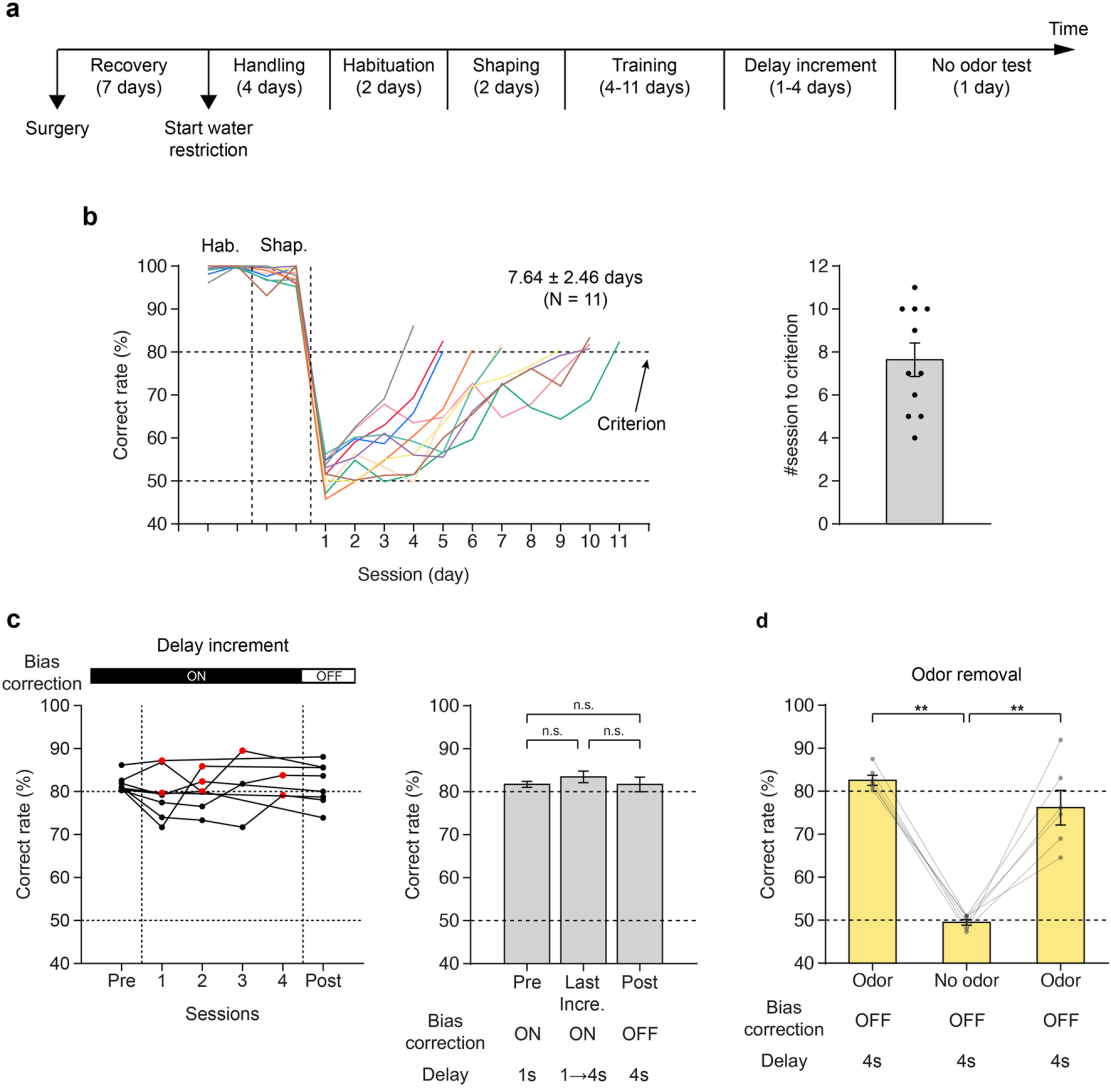
Performance of the olfactory DMS task. (**a**) Overall experimental timeline. (**b**) Left: Learning curves showing the correct rate. The different colors represent different subject mice. Right: Number of training sessions required to reach the performance criterion (80%). (**c**) Left: Changes in the mean correct rate across delay increment sessions. Pre refers to the session in which the subjects exceeded the mean correct rate of 80% for the first time. Post indicates the mean correct rate for the session with a fixed delay period of 4 s from the start without bias correction. Red dots indicate the last delay increment sessions for each subject. Right: Mean correct rate (across sessions) before the delay increment phase (Pre), for the last delay increment session (Last incre., red dots in the left panel), and after the delay increment session (Post) (*n* = 8 mice, mean ± SEM). (**d**) Odor-removal experiment to exclude the possibility of the involvement of visual, auditory, and somatosensory cues (*n* = 6 mice, mean ± SEM).

Our training protocol began with a pre-training phase, which involved 4 days for handling, 2 days for habituation, and 2 days for shaping. The subsequent training phase took 4–11 daily sessions, depending on the performance of the mice (Fig. 3a). A subset of mice (*n* = 8) underwent further training with an increasing delay period (delay increment phase). The details of this training are presented below.

#### Handling

After at least a week of recovery from surgery, the mice were subjected to a water restriction schedule (0.8–1 mL water/day; ~ 80% of the normal body weight). After 3 days of handling, during which the mice were gently held in the experimenter’s hands for 10 min each day, they were mounted on the head-fixing device in the behavior box and were provided drops of water (2 μL) through the left and right lickports by manually clicking the “water reward” button on the custom-made GUI. Because the mice were not yet familiar with the locations of the left and right lickports, the experimenters adjusted the positions of the lickports, bringing them closer to the mouth by clicking the “ −1 step” button on the GUI. Once the mice began licking, the experimenters gradually moved the lickports away, one step at a time. The subject mice became familiarized with the locations of the left and right lickports and learned that they could obtain water rewards by licking either of the lickports within a ten-minute period.

#### Habituation

The habituation phase aimed to establish an association between the ascending sound of the linear actuator and the commencement of the response window. Before a trial started, both lickports were in the inactive position. When the trial commenced, one of the two lickports ascended to the active position. A water reward was provided when the subject mice made contact between their tongue and the tip of the lickport in the active position. Once the trial ended, the active lickport returned to the inactive position. This phase spanned 2 days, employing a block-wise approach. On the first day, alternating sequences of five left-rewarded and five right-rewarded trials were presented. On the second day, alternating sequences of three left-rewarded and three right-rewarded trials were used. To minimize the occurrence of missed trials, the response window was set to 30 s. When necessary, the experimenter adjusted the position of the lickports, either moving it closer to or further away from the mouse’s mouth, to encourage licking behavior. The session ended once the mice had obtained 200 water rewards.

#### Shaping

This phase aimed to familiarize the mice with the temporal structure of the DMS task. Two odor stimuli were presented sequentially with a 1-s delay between them. One second after the offset of the second odor stimulus, only the lickport associated with the reward (left for nonmatch trials and right for match trials) was moved to the active position at the beginning of the response window, while the other lickport remained in the inactive position throughout the trial. One out of four different combinations of stimuli (EA-2P, 2P-EA, EA-EA, or 2P-2P) was randomly chosen for each trial. The mice completed approximately 200 trials during this phase, which lasted for 2 days.

#### Training

In this phase, the mice were trained to lick the left lickport when two sequentially presented odors were identical and the right lickport when the two odors differed (see **Delayed match-to-sample task** section). Unlike in the shaping phase, both lickports were moved to the active position at the beginning of the response window, regardless of the trial condition. The automated side bias correction algorithm (see **Automated side bias correction algorithm** section) was employed during this phase, resulting in the active position of the lickports being adjusted on a trial-by-trial basis using both long-term and short-term corrections. The delay period between the presentation of two stimuli was set to 1 s.

In each trial (trial *k*), the trial conditions (nonmatch or match) for the next ten trials were predetermined in a random order. The proportion of left-rewarded trials (${P}_{L}(k)$) was 0.5 for the initial 30 trials, and was then adjusted by the long-term correction for the subsequent trials. For each trial in the sequence, the first stimulus odor was randomly selected, and the second stimulus odor was assigned based on the trial condition. This procedure was repeated every ten trials as long as the proportion of left-rewarded trials (${P}_{L}(k)$) remained unchanged. Whenever ${P}_{L}(k)$ changed, the sequence of match and non-match trials for the next ten trials were determined again. The training phase ended once the mice achieved a correct rate > 80% for the session. The mice performed approximately 300 trials (~ 1.5 h) per day.

### Delay increment

This additional phase was designed for researchers requiring a longer delay period in their experiments. When the delay period abruptly and significantly changes, mice may require additional training to adapt to the altered temporal structure of the task. To minimize the unnecessary prolongation of training resulting from this change, we implemented a 50-ms increment in the delay period per trial based on the mice’s performance. Specifically, if both the correct rate for the most recent ten nonmatch trials and ten match trials was > 80%, the delay period was increased by 50 ms. This incremental adjustment continued until the delay period reached 4 s. The phase ended once the animals consistently achieved correct rates of > 80% for at least 100 consecutive trials with the target delay of 4 s. When this criterion was not met within a single session, the phase was restarted the next day with an initial delay period of 1 s rather than continuing from the delay period achieved on the previous day. After completing the delay increment training, the mice underwent an additional session with the bias correction algorithm turned off.

### Odor-removal experiment

An odor-removal experiment was conducted to eliminate the possibility that mice performed the task relying on other sensory cues from the task devices, such as the sound produced by the solenoid valve and the linear actuator. The odor-removal experiment session consisted of three blocks of trials. In the first block, mice performed the olfactory DMS task for at least 50 trials (71 ± 10.80 trials, *n* = 6 mice). If the subject mice did not achieve a performance ≥ 80% within a maximum of 100 trials, they did not proceed to the odor-removal block. If they did, the session was temporarily paused to remove the odorants from the olfactometer. Further, the session resumed for the second block of at least 50 trials (69 ± 18.98 trials), followed by the third block in which the odorants were added again (104 ± 33.01 trials). The automated side bias correction algorithm was not applied during the odor-removal experiment.

### Analysis of behavioral performance

Unless otherwise stated, all data analyses were conducted using custom MATLAB codes (2019a, Mathworks, USA). The correct rate was determined by dividing the number of correct trials by the total number of trials.

### Quantifying the side bias

**Figure 4:**
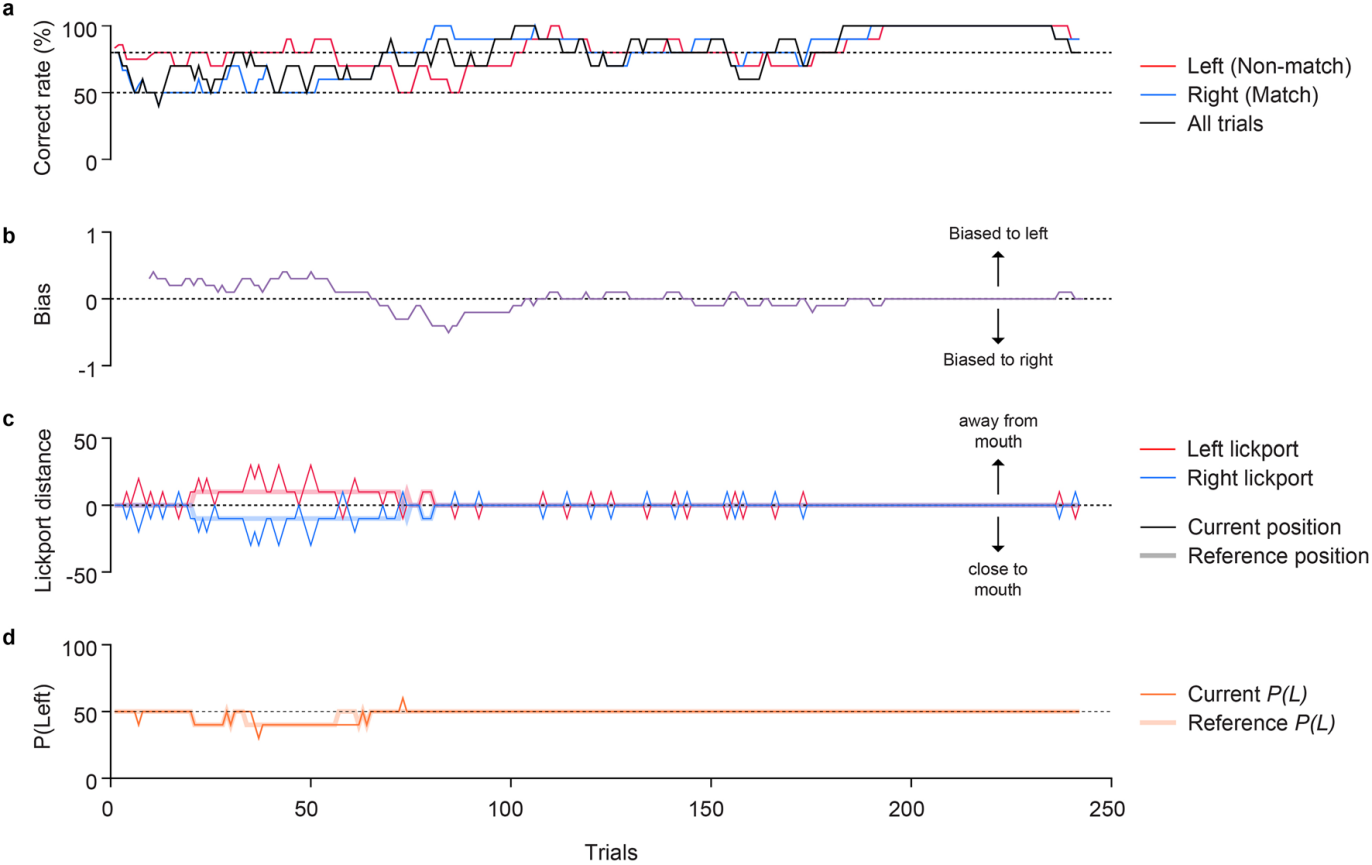
Example behavioral traces in a single session. (**a**) Changes in correct rates (10-trial moving average) in left (red), right (blue), and all trials (black). (**b**) Changes in side bias. (**c**) Changes in the lickport distances. Thin solid lines represent the physical positions of the lickport (${D}_{L}(k)$ and ${D}_{R}(k)$), whereas the thick translucent lines represent the reference positions ($({D}_{L}^{ref}(k)$ and ${D}_{R}^{ref}(k)$). The initial reference positions (${D}_{L}^{ref}(1)$ and ${D}_{R}^{ref}(1)$) are set to zero. A positive value indicates lickport farther from the mouth than the reference position, whereas a negative value indicates closer to the mouth. (**d**) Changes in the proportion of left trials. The thin solid line indicates the current proportion of left trials. The thick translucent lines represent the reference proportion of left trials.

The side bias (Fig. 4b) in trial *k* was calculated as shown below:

$$\frac{{n_{corr\_left} \left( k \right)}}{{n_{left} \left( k \right)}} - \frac{{n_{corr\_right} \left( k \right)}}{{n_{right} \left( k \right)}}$$

where ${n}_{left}(k)$ and ${n}_{right}(k)$ represent the number of left-rewarded (non-match) and right-rewarded (match) trials among the last 20 trials, and ${n}_{corr \_left}(k)$ and ${n}_{corr \_right}(k)$ are the number of trials in which the subject mice chose the correct lickports in the left and right trials, respectively, among the last 20 trials. The side bias was evaluated after the initial 20 trials of the session.

### Statistics

Statistical differences were determined using two-sided paired t-tests or paired Wilcoxon signed-rank tests. A *p* value < 0.05 was considered statistically significant. All analyses were performed using MATLAB (2019a, Mathworks, USA). All data were presented as mean ± standard error of the mean (SEM).

## Results

### Fast learning of the DMS task with side bias correction algorithm

By implementing our correction algorithm for lickport positions, all subject mice (*n* = 11) achieved ≥ 80% accuracy within a few sessions (7.64 ± 2.46 sessions; Fig. 3b). The training time was substantially shorter than that in a previous study that used a similar behavioral paradigm but did not employ a side bias correction algorithm^20^.

DMS tasks are frequently employed to investigate working memory, which involves the ability to retain information regarding the first stimulus during a delay period until the presentation of the second stimulus. As the delay between the presentation of the first and second stimuli increases, the DMS task becomes more challenging for animals to perform. Typically, animals are initially trained with a short delay and gradually exposed to longer delays to acclimate them to the task. Thus, we evaluated the effectiveness of our side bias correction algorithm in extending the delay between two stimuli in the DMS task.

Once the subject mice attained a correct rate ≥ 80% in the initial training phase with a one-second delay, eight of them proceeded to the delay increment phase. In this phase, the delay was systematically increased by 50 ms after each trial as long as the mouse maintained a correct rate ≥ 80%. The delay increment phase ended when the delay reached 4 s, and the mice attained a correct rate ≥ 80% for at least 100 consecutive trials (Fig. 3c; See ‘Methods’ section). Remarkably, the mice completed this phase within 1–4 sessions (mean, 2.38 ± 1.19 sessions; Fig. 3c, left). Moreover, the behavioral performance did not show significant changes across sessions (Last training session with a one-second delay (Pre) versus Last delay increment session (Last Incre.) in Fig. 3c, right, *t*_*7*_ =  − 1.54, *p* = 0.17).

Next, we examined whether the mice could maintain their performance without the bias correction algorithm. When the bias correction algorithm was turned off, the behavioral performance of the mice did not differ significantly from that in the last delay increment session (Last Incre. vs. Post in Fig. 3c, right, *t*_*7*_ = 2.17, *p* = 0.07), as well as the performance in the last training session with a one-second delay (Pre vs. Post in Fig. 3c, right, *t*_*7*_ = 0.01, *p* = 0.99). These results suggest that once mice are well-trained using the bias correction algorithm, it is no longer necessary for them to continue performing the DMS task.

For a subset of the mice (*n* = 6), we further tested whether they relied on the olfactory cues to perform the task (Fig. 3d). When fresh air was delivered instead of the odor cues during the cue presentation periods, the correct rate dropped approximately to 50%. However, when the odor cues were reintroduced, the performance of the mice immediately recovered. These results suggest that the mice performed the DMS task by comparing the presented odor cues.

### Changes in behavioral performance and bias correction parameters during DMS task training

A typical example of a session exhibited notable discrepancies in correct rates between the left- and right-rewarded trials (Fig. 4a). Specifically, the mouse demonstrated a high correct rate on its preferred side and a low correct rate on the nonpreferred side (Fig. 4a). These periods coincided with significant licking biases displayed by the mice (Fig. 4b). To address these apparent biases, our correction algorithm intervened by adjusting the distance between the lickports and the mouse’s mouth, as well as the proportions of the left- and right-rewarded trials (Fig. 4c, d). As the bias diminished, the lickport positions gradually returned to the original symmetric configuration (Fig. 4b, c). Furthermore, once the bias was mitigated, the correct rates exhibited a marked improvement and remained high (Fig. 4a).

Overall, these observations strongly indicate that our correction algorithm was highly effective in reducing side bias and, consequently, improving the correct rate. Thus, we proceeded to quantitatively assess the efficacy of our correction algorithm.

### Rapid reduction of the side bias during DMS task training

**Figure 5:**
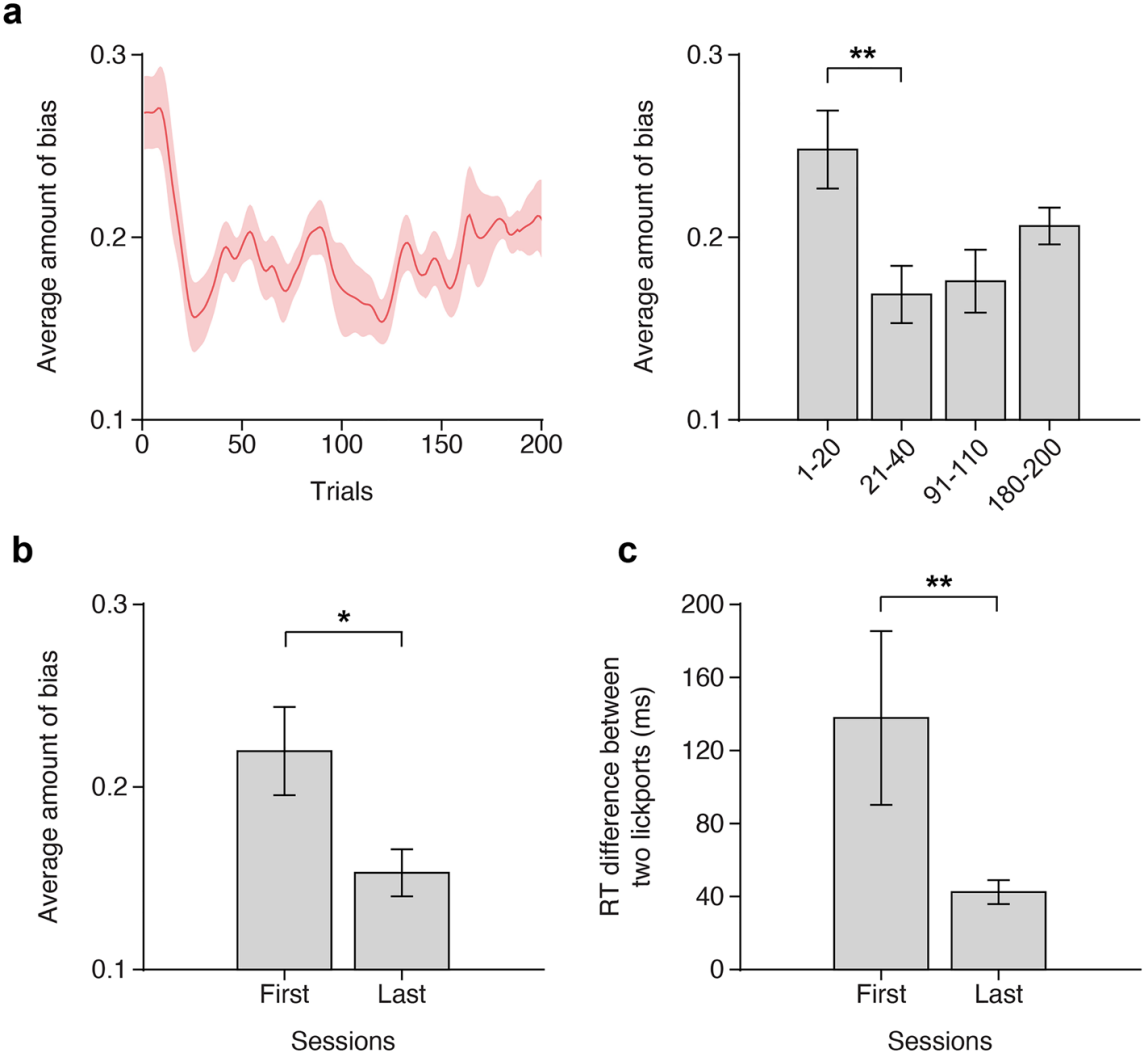
Alleviation of side bias within and across training sessions. (**a**) Side bias within a single session. Left: Changes in bias. Mean ± SEM (shaded area). Right: Mean side bias of the first, second, middle, and last 20 trials of sessions. (**b**) Side-bias in the first and last DMS training sessions. (**c**) Difference in the mean response time (RT) between two lickports in the first and last DMS training sessions.

To investigate the efficacy of our correction algorithm in mitigating side bias, we closely examined the changes in side bias throughout the training. We first examined how the side bias changed within a single session. We noticed that the mice exhibited a higher level of side bias at the beginning of each session, which rapidly diminished in the subsequent trials (Fig. 5a left). Notably, a significant decrease in side bias was observed within the first 40 trials (Fig. 5a right, One-way ANOVA: *F*(3, 36) = 4.74, *p* = 0.0069; the first 20 trials versus the next 20 trials: *t*_*9*_ = 4.34, *p* = 0.0019). Furthermore, the side bias decreased across sessions as training progressed (Fig. 5b; *t*_*9*_ = 2.43, *p* = 0.0379). These rapid alleviations of side bias within and across sessions seemed to expedite the training process.

Additionally, we observed that the response time for the lickport on the nonpreferred side was longer than that on the preferred side. However, this discrepancy in the response time between the left and right lickports considerably diminished as training advanced (Fig. 5c; *p* = 0.0059, two-sided paired Wilcoxon signed-rank test).

These findings strongly indicate that our automated correction system rapidly reduced side bias. This reduction is likely associated with a notable improvement in behavioral performance.

### Rapid reduction of the side bias via the adjustment of lickport distances and trial proportions

Next, we investigated the effects of the adjustment of lickport distances and the manipulation of left- and right-rewarded trial proportions on side bias.

**Figure 6:**
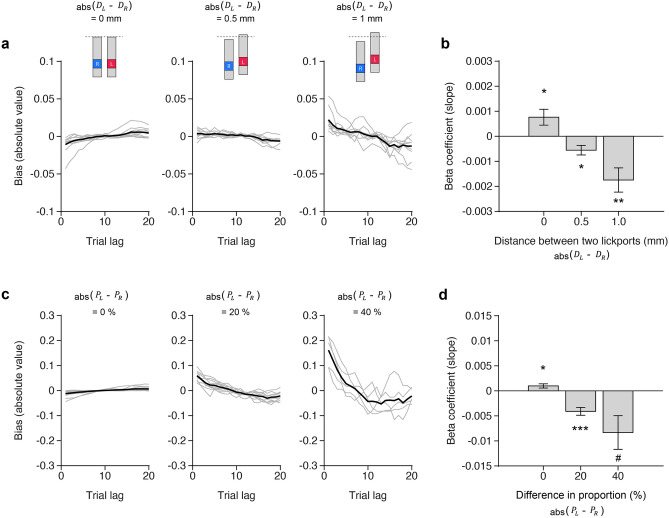
Effects of adjusting lickport positions and trial proportions on side bias. (**a**) Changes in side bias during the next 20 trials following trials with specific differences in lickport distances (left: 0, middle: 0.5, and right: 1.0 mm). (**b**) Beta coefficient (slope) of linear regression for the changes in side bias in (c). (**c**) Changes in side bias during the next 20 trials following trials with specific differences in trial proportions (left: 0%, and right: 20%). Only trials with 0% and 20% were analyzed owing to the small number of trials in other difference values. (**d**) Beta coefficient (slope) of linear regression for the changes in side bias in (**e**).

First, we examined how the difference in the distances to the two lickports affected side bias. When the lickports were positioned at an equal distance from the mouse’s mouth, the side bias tended to increase during the following 20 trials (Fig. 6a, left; Fig. 6b, *t*_*9*_ = 2.53, *p* = 0.03). However, when the difference in the distances to the lickports was 0.5 and 1 mm, the side bias showed a tendency to decrease during the subsequent 20 trials (Fig. 6a, middle and right; Fig. 6b, 0.5 mm: *t*_*9*_ =  − 3.07, *p* = 0.013, 1 mm: *t*_*9*_ =  − 3.82, *p* = 0.005). These results indicate that placing the preferred lickport farther away from the mouth and the nonpreferred lickport closer to the mouth effectively reduces the side bias of mice.

We also investigated the effect of the disparity in trial proportions between left- and right-rewarded trials on side bias. When the left- and right-rewarded trials were presented with equal probabilities, we observed an increase in side bias over the following 20 trials (Fig. 6c, left; Fig. 6d, *t*_*9*_ = 2.54, *p* = 0.0318). However, when there was a 20% or 40% difference in the proportions of the two conditions, we found that the side bias decreased in the subsequent 20 trials (Fig. 6c, middle and right; Fig. 6d, 20%: *t*_*9*_ =  − 6.06, *p* = 0.0002, 40%: *t*_*4*_ =  − 2.7681, *p* = 0.0504). These findings indicate that presenting fewer trials for the preferred side and more trials for the nonpreferred side effectively decreased the side bias in mice.

### Improvement of correct rate via the adjustment of lickport distances and trial proportions

Finally, we aimed to assess the effects of adjusting lickport distances and trial proportions on correct rate.

**Figure 7:**
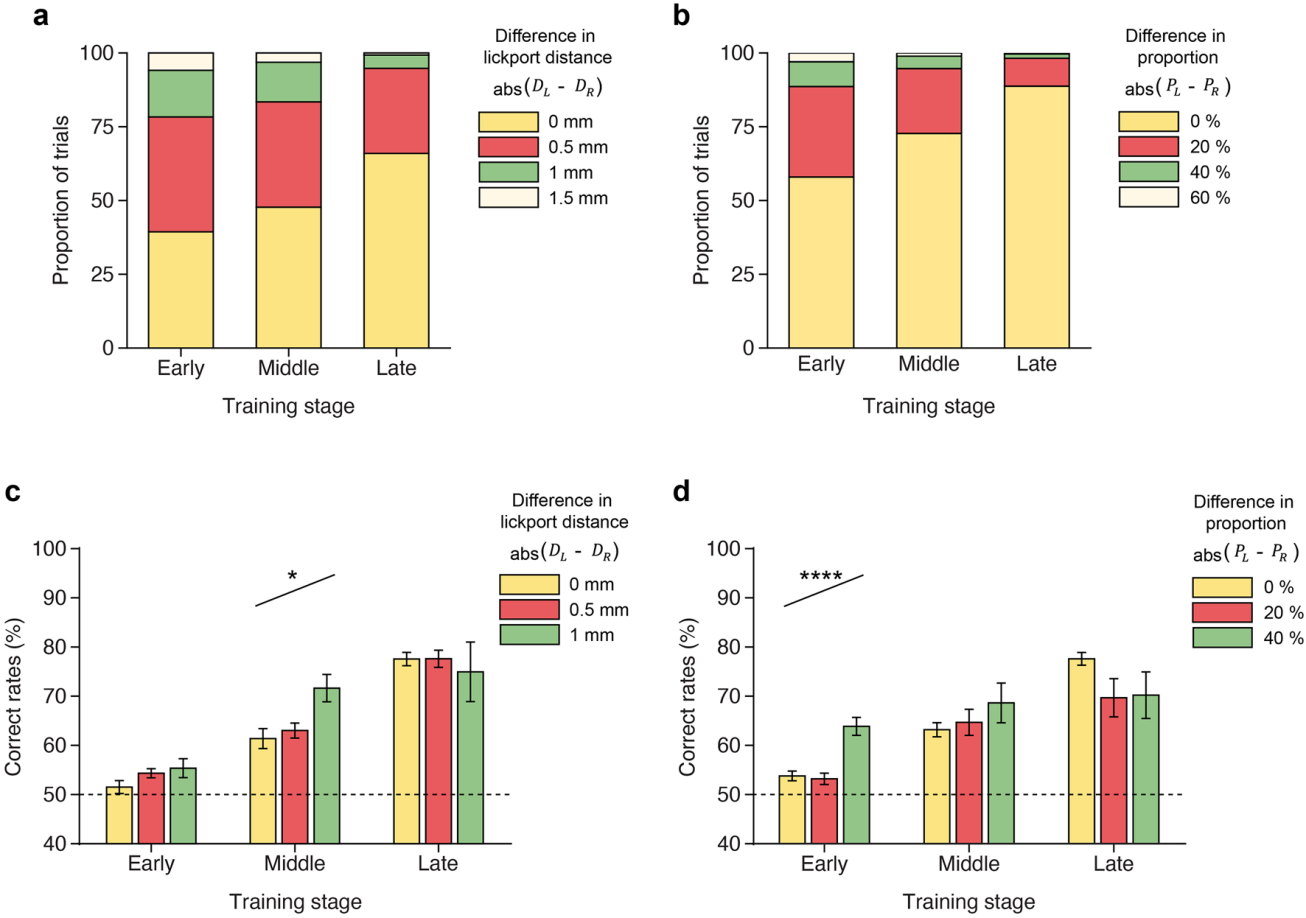
Effects of adjusting lickport positions and trial proportions on behavioral performance. (**a**) Proportions of trials with varying differences in the distances between the left and right lickports in different training stages. (**b**) Proportions of trials with varying proportion differences between left- and right-rewarded trials in different training stages. (**c**) Correct rates for trials with varying differences in lickport distances in different training stages. (**d**) Correct rates for trials with varying differences in the proportions of left- and right-rewarded trials in training stages.

First, we examined the proportions of trials with varying differences in the distances between the left and right lickports. As the lickport positions were adjusted, the left and right lickports moved simultaneously in opposite directions by 0.25 mm per trial. Consequently, the difference between the two lickport positions became a multiple of 0.5 mm. The majority of trials exhibited a difference ≤ 1 mm, corresponding to two steps away from equidistance (Fig. 7a). This result indicated that increasing the difference rapidly improved behavioral performance, leading to a subsequent decrease in the difference back toward zero. Moreover, the proportions of trials with different difference-in-distance values changed throughout training. Specifically, the proportion of trials with a 1-mm difference progressively decreased, whereas the proportion of trials with a 0-mm difference progressively increased (Fig. 7a). Similarly, the discrepancy in proportions between left- and right-rewarded trials was more pronounced in the early training sessions, as indicated by larger proportions of the trials with a 40% difference and smaller proportions with a 0% difference (Fig. 7b). As training progressed, the proportion of trials with a 20% or 40% difference decreased, whereas the proportion of trials with a 0% difference increased (Fig. 7b).

Next, we investigated whether the adjustments of lickport distances enhanced behavioral performance by computing correct rates for trials with difference values of 0, 0.5, or 1 mm across different training phases (Fig. 7c). We found that the difference in distances between lickports did not show a correlation with the correct rate in the early training phase (Early: *t*_*10*_ = 1.85, *p* = 0.0966, Bonferroni-corrected based on three tests). Similarly, no correlation was observed in the late training phase (Late: *t*_*10*_ =  − 0.50, *p* = 0.6301, Bonferroni-corrected). However, a significant correlation between the difference in distance and the correct rate emerged during the middle training phase (Middle: *t*_*10*_ = 3.12, *p* = 0.0116, Bonferroni-corrected).

Lastly, we explored the influence of adjusting the proportions of the left- and right-rewarded trials on correct rate. We found that a 40% difference in trial proportions had a more pronounced effect on the correct rate in the early training phase compared to smaller differences in trial proportions (Fig. 7d, Early: *t*_*10*_ = 5.07, *p* = 0.0007, Bonferroni-corrected based on three tests). However, the correlation between the difference in trial proportions and the correct rate became less evident and even reversed in the later phases of training (Fig. 7d, Middle: *t*_*10*_ = 1.30, *p* = 0.2266, and Late: *t*_*10*_ =  − 1.34, *p* = 0.2158, Bonferroni-corrected). These results suggested that a substantial difference in the proportions of left and right trials aids in the mice’s performance during the early training phase.

Because our side bias correction algorithm adjusted both lickport distances and left- and right-rewarded trial proportions, it is challenging to dissociate their effects on task performance. To examine the pure effect of each adjustment, we analyzed the trials where only one of them was altered while the other remained not adjusted. We found that the proportions of the left- and right-rewarded trials was not adjusted in 73.46% ± 10.81% of all trials. When we reanalyzed the effect of adjusting lickport distances on correct rate for these trials, a significant effect was still observed in the middle training stage (Early: *t*_*9*_ = 1.29, *p* = 0.2292; Middle: *t*_*9*_ = 3.63, *p* = 0.0055; Late: *t*_*9*_ =  − 1.01, *p* = 0.3379). This result suggests that adjusting lickport distances alone could improve the correct rate of the DMS task. However, we could not examine the pure effect of adjusting left- and right-rewarded trial proportions in a similar way because the trials in which the proportions of left- and right-rewarded trials were adjusted while the lickport distances remained identical were only 3.20% ± 1.00% of all trials.

## Discussion

DMS tasks conducted with head-fixed animals provide excellent paradigms for investigating sensory processing, working memory, and decision-making processes. Moreover, these tasks offer the advantage of easy integration with imaging, electrophysiological recordings, and neural activity manipulation techniques. Although DMS tasks using a single lickport for asymmetric Lick/No-Lick choices introduce uncertainties in interpreting licking responses^7–9^, they have been used more frequently than tasks with two-alternative lickport choices on the left and right side because the former requires significantly shorter training time^20,49,50^. In our study, we made substantial improvements in reducing the training time for a DMS task that incorporates two-alternative lickport choices. The average training time required for mice to attain a correct rate > 80% was approximately 1 week (Fig. 3b), which is substantially faster than that reported in the previous study conducted under similar experimental settings, except for the use of an automatic side bias correction^20^. We accomplished this by dynamically adjusting the lickport positions and manipulating the proportions of left- and right-rewarded trials using a side bias correction algorithm.

While both the long-term and short-term correction algorithms were designed to alleviate side biases, they were implemented differently. The long-term correction algorithm did not directly adjust lickport positions and trial ratios. Instead, it sets reference positions for the lickports and reference trial ratios, which served as targets for the short-term adjustment. Because the extent of the long-term adjustments of reference lickport positions and reference trial ratios was determined based on the side bias observed throughout the entire session, it addressed the change in the overall level of side bias. In contrast, the short-term correction algorithm made immediate adjustments to counteract transient fluctuations in side biases. Specifically, when an incorrect lickport choice occurred, it moved the chosen lickport farther away and brought the other lickport closer. However, after a correct lickport choice, both lickports were moved toward their reference positions. This approach aimed to increase the difference between two lickport distances when the mice frequently made incorrect choices on one lickport due to a temporary increase in side bias. On the other hand, the trial ratio adjustment by the short-term correction algorithm only occurred after three consecutive incorrect choices for the same lickport, which indicated a more substantial side bias.

Our analyses demonstrated that the adjustments in lickport distances and left- and right-rewarded trial ratios effectively reduced side bias in the subject mice. Although it is challenging to isolate the individual effects of lickport position adjustments and trial ratio manipulation, we observed that the extent of the difference in lickport distance and the trial proportions tended to correlate with the correct rates in the early and middle training stages, indicating a positive effect on the improvement of the correct rate. Interestingly, these adjustments appeared to be more effective in different training stages. Specifically, the difference in trial proportions had a strong effect in the early training stage, whereas the difference in lickport distances was more effective in the middle training stage. This suggests that effective training strategies differ between these training phases. When the mice are in the initial training stage, the disparity in trial proportions, which allowed for more frequent presentations of either left- or right-rewarded trials, appeared to aid the mice in associating the trial type with the corresponding lickport. Indeed, a similar strategy has been used in previous DMS trainings, where a shaping phase involved repeated presentation of the same trial type to help the animals make proper associations between the stimulus combinations and lickports^49,50^. In contrast, during the middle training phase, we observed that a larger difference in lickport distance was related to a higher correct rate. This suggests that as learning progresses, the minimization of the influence of licking bias becomes more effective in improving their performance. In the late training stage, both the adjustments of lickport distances and trial proportions did not show a significant effect on behavioral performance. This is likely because of the low side bias and the high behavioral performance in this stage. However, it is worth noting that a substantial proportion of trials still displayed a 0.5 mm difference in the distances to the lickports. This observation suggests that the lickport adjustment may still exert a positive effect in the late training stage by preventing the re-establishment of side bias.

A shorter training duration reduces the workload associated with training and offers additional advantages. First, it provides a better opportunity to track the activity of the same neurons throughout the entire learning process in the DMS task. Although imaging approaches enable the tracking of a neuron’s activity over a long period, the efficiency of tracking decreases as the time interval between imaging sessions increases. Thus, a short training duration increases the likelihood of monitoring changes in the activity of the same neurons during the entire learning process. Second, our automated lickport adjustment system enables us not only to mitigate side bias but also to artificially introduce side bias. This versatile manipulation of side bias can be harnessed to reveal neural mechanisms underlying choice bias itself. Last, the system can be applied to Go/No-Go tasks with a single lickport^49^. In a Go/No-Go paradigm, animals typically display a preference for licking rather than refraining from licking. This preference often leads to licking responses in both Go and No-Go trials during the early training sessions, impeding the rapid learning of task. This preference can potentially be counterbalanced by positioning the lickport far from the animal’s mouth in No-Go trials.

In our training protocol, the experimenter initially determines the reference positions for the lickports where the mice can comfortably lick both sides. During the training process, the lickport positions are adjusted using the side bias correction algorithm. Upon the training completion, the lickports eventually return to their initial reference positions as set by the experimenter. However, it remains uncertain whether these initial lickport positions are optimal for each individual mouse to conveniently lick both left and right lickports equally. Mice can have varying oral structures, and some may find it more comfortable to extend their tongues farther toward the left side. Consequently, if the left and right lickports are equidistant from their mouths, the mice may display a bias toward the left side. In such cases, adjusting the position of the right lickport to a closer location would ensure that subject mice feel equally comfortable when licking both lickports. While our correction algorithm currently relies on the experimenter’s subjective estimation for the optimal lickport positions, future endeavors to enhance the side bias correction algorithm may include the development of algorithms that identify subject-specific optimal lickport positions. These algorithms would aim to minimize side bias and maximize performance by considering the unique characteristics and preferences of individual animals.

## Data Availability

The datasets used and/or analyzed during the current study available from the corresponding author on reasonable request.
